# Fast File Transfers from IoT Devices by Using Multiple Interfaces

**DOI:** 10.3390/s21010036

**Published:** 2020-12-23

**Authors:** Leonardo Mostarda, Alfredo Navarra, Francesco Nobili

**Affiliations:** 1 Computer Science Department, University of Camerino, 62032 Camerino, Italy; 2 Mathematics and Computer Science Department, University of Perugia, 06123 Perugia, Italy; alfredo.navarra@unipg.it; 3 Chemistry Department, University of Camerino, 62032 Camerino, Italy; francesco.nobili@unicam.it

**Keywords:** smartphone, multi-interface network, file transfer, WiFi, Bluetooth, 4G

## Abstract

The Internet-of-Things (IoT) is a modern technological revolution that enables communication amongst a plethora of different devices. To date, about 30 billion devices have been connected to the internet and more than 75 billion devices are probably to be connected worldwide by 2025. These can range from small sensors and actuators to larger devices such as smartphones, drones or even buildings and interconnected cars. Devices are often mobile and battery powered thus their communication requires fast and energy efficient solutions. To this end, this paper studies the use of multi-interface communication for fast and energy efficient communication. In particular, we consider the basic operation of data transfer between smartphones in the form of files. This task can be performed for backup purposes, and hence it represents a useful and frequent operation that users perform. Our aim is to provide a new and easy means that optimises file transfers with respect to time and energy consumption. In particular, as smartphones are endowed with various connecting interfaces like Bluetooth, WiFi and 4G, we conduct experimental studies by varying different parameters in order to understand the best setting, including which interface is more appropriate to accomplish file transfer. To this respect, we also implemented an innovative and light app that allows the use of two or more interfaces concurrently. The experimental results show how the coupling of some interfaces might be effective in terms of time, while consuming a negligible amount of energy. Actually, such results become more and more interesting as the size of the file to be transferred grows. The best combination experienced is by making use of WiFi at 5 GHz concurrently with 4G, whereas WiFi at 2.4 GHz caused interference complications.

## 1. Introduction

The Internet-of-Things (IoT) is composed of interconnected computing machines, mobile devices, sensors and actuators with unique identifiers that have the capability of sending data over a network without the need for human interaction [1,2]. These can range from small sensors and actuators to larger devices such as smart phones, buildings, drones and interconnected cars. Devices are often mobile and battery powered, thus their communication requires fast and energy efficient solutions. To this end, this paper studies the use of multi-interface communication that is the interconnection of devices by using heterogeneous interfaces. Multi-interface communication takes advantage of modern devices which are usually equipped with various interfaces such as 4G, Bluetooth and WiFi. A number of applications are beginning to exploit multi-interface networks to interconnect the heterogeneous devices. For instance, the Siri Apple service monitors the quality of WiFi and cellular networks and automatically switches between them. This allows the service to improve its bit rate and response time. In general, the selection of the best interfaces for device interconnection can be affected by various factors. For instance, the interface features such as bandwidth, energy consumption and bit-rate constitute some relevant elements. Furthermore, the neighbourhood of a device and whether or not an interface is available on some devices clearly affect the possibility to communicate via a specific interface. IoT devices are often mobile or portable. This requires interconnection solutions which prolong the lifetime of the established network and allow fast communication. In fact, a slow transmission rate can limit the node mobility speed. Energy inefficient interconnections can cause device failure as a consequence of battery depletion. This challenging optimisation problem must consider different parameters (i.e., energy and communication speed) at the same time. Energy can be the primary focus when the IoT devices cannot be replaced while speed can be vital for frequent operations such as backup where the user would need to quickly finish the task.

In this paper, we study a very simple and basic task that is file transfer from a smartphone, potentially to any other device that shares at least one common interface. Our aim is to understand if optimisation with respect to energy and time can be obtained by suitably selecting the communication interface or most importantly by concurrently combining two or more interfaces into a single connection.

### 1.1. Motivation

The main reason why we started working on multi-interface networks is due to the evident availability of multi-interfaces in devices. Surprisingly, exploiting such interfaces in a concurrent way has not been investigated nor implemented so far. Hence, the questions we posed are:

- Can it be the case that nobody as thought about such a scenario so far?
- How much difficult can it be from a technological view point to implement concurrency among the interfaces?
- Is the lack of investigation with respect to practical multi-interface networks due to the actual inefficiency of implementing concurrent solutions?
- Can we optimise data rate as well as energy consumption?

From the literature, only theoretical aspects along with algorithmic complexity have been highlighted and deeply investigated but no practical and real experimentation has been conducted. Indeed, our questions are rather natural and they certainly deserve investigation.

Hence, the first scenario we have imagined concerns a very standard, useful and practical operation, that is *file transfer*. This is one of the main tasks we require in smart devices for accomplishing many different activities. We aim to investigate how efficiently the file transfer can be performed by means of wireless connections, possibly exploited concurrently. To this purpose, we also need to investigate whether this is technologically feasible with respect to various producers. In particular, we consider as first attempt the Android system which is certainly an open environment supported by many forums and documentation. Our purpose is then to design a new app for Android devices that allows the user to activate multiple interfaces concurrently in order to accomplish the same file transfer. In practice, when more than one interface is used, the file to be transferred must be split into different chunks that are opportunely subdivided among the active interfaces. The choice of the interfaces as well as the size and ratio of the chunks assigned to a specific interface are all parameters that should be carefully managed and possibly automatically configured.

### 1.2. Our Results

As we are going to show, we successfully overcome all technological barriers within the Android system. In fact, we devise a new app that can be installed over the Android system and that can be used to suitably activate the available interfaces. The app also provides an automatic mode that allows the user to not bother with the percentage of file chunks to be assigned to a specific interface. This can be done manually, but we also implement an automatic mode that in a dynamic way assigns chunks to the interfaces proportionally to the current quality (in terms of bandwidth) available for the used interfaces. We then conduct a very accurate and cautious experimentation for the file transfer task by varying many parameters, such as the file size, specific device, combination of different interfaces or their stand-alone performance. The practical outcome reveals how the coupling of some interfaces might be effective in terms of time, while consuming a negligible amount of energy more. The best combination experienced is by making use of WiFi at 5 GHz concurrently with 4G, whereas with WiFi at 2.4 GHz, we faced interference complications. Actually, the obtained results become more and more interesting as the size of the file to be transferred grows.

### 1.3. Outline

The rest of the paper is organised as follows. Section 2 reviews the state-of-the-art of multi-interface networks and their application. In Section 3, we provide all the ‘ingredients’ required for our experimental studies on file transferring from smartphones. More precisely, we specify all characteristics of the smartphones we have used in our experiments and their operating systems. We provide details on how time and energy consumption have been evaluated during the file transfers. We describe a new and light Android app we have developed for managing the usage of different interfaces in their stand-alone or concurrent settings. Section 4 contains all data, comments and results that are related to the experimental results experienced during our studies on file transfers from smartphones. Finally, Section 5 provides useful discussions to further investigate this practical and intriguing context.

## 2. Related Work

The authors in [3] study the transferring of files when WiFi or Bluetooth interfaces are used. Unlike our approach, their experiments do not transfer files by using WiFi and Bluetooth concurrently. The authors demonstrate that, when small-size files need to be sent, Bluetooth has better performance than WiFi, whereas for larger files, WiFi is always a better choice. The experiments were performed by using different hardware equipped with different operating systems.

The authors in [4,5] study the concurrent use of WiFi and LTE interfaces. They focus on their communication interference and various threads scheduling problems at an operating system level. Unlike our approach, they do not measure the performance in terms of energy and throughput for sending and receiving data by the interfaces at the same time. In [6] the authors try to optimise the file transfer on mobile phones by using 3G and WiFi. While they provide interesting results, they do not study the use of both 3G and WiFi at the same time.

The multi-path TCP (MTCP) [7] is an interesting industrial field which is related to our research. MTCP advocates the use of communications that uses multiple TCP paths. While MTCP works at the transport layer, our research focuses on the application layer (i.e., it is independent from any networking and transport layers). Some of the conclusions that are proper of the MTCP are also observed in our research. More precisely, the use of simultaneous communication over multiple interfaces improve throughput and fault tolerance. However, to the best of our knowledge, no MTCP study that focuses on energy consumption has been performed on smartphones. In fact, most smartphone operating systems do not support MTCP. This led us to our application layer study on multi-interface communications where we could perform all the required experiments. The application level implementation also allows to overcome the problem of the internet middleboxes (e.g., firewalls and NATs) that greatly complicate the applicability of MTCP. The reader interested to MTCP and related techniques may refer to [8,9].

In this paper, we are interested in uploading operations from smartphones, as we consider the data collection as reference task. We want to exploit the available hardware as much as we can, hence also allowing the file transfer via multiple interfaces, that is splitting the file into chunks that are distributed among the available connections. Approaches related to this aim have been considered in [10,11], even though the lack of technical details allows little room for reproduciblity. For instance, even the specification of which devices have been tested for the experimentation is missing, or the way energy consumption has been measured.

**Figure 1:**
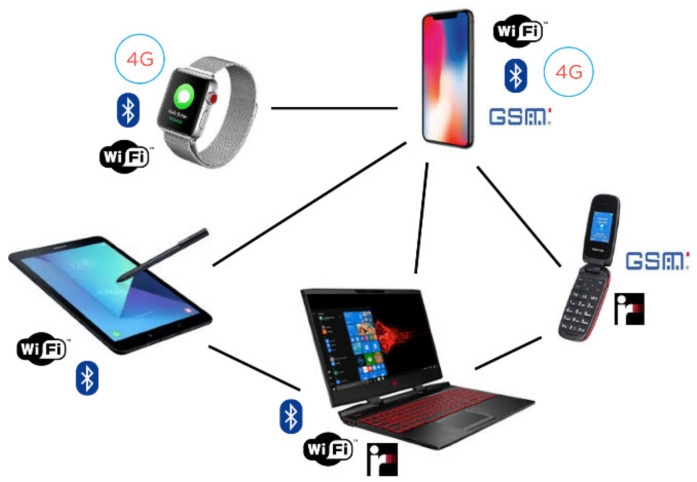
Example of multi-interface network composed of heterogeneous devices connected by means of different interfaces.

So far, the study of the so-called *Multi-Interface* networks has been mainly performed from a theoretical prospective. In general, a network of devices is described by a graph G=(V,E), where *V* represents the set of devices and *E* is the set of possible connections defined according to the distance between devices and the available interfaces that they share. Each v∈V is associated with a set of available interfaces W(v). All possible interfaces are defined by the set ${⋃}_{v\in V}W\left(v\right)$ that has cardinally *k*. A connection is established when the endpoints of an edge have at least a common interface that is active. When a node *u* has an interface *I* activated, *u* consumes an amount of energy equal to c(I). This is used in order to maintain *I* active. The interface *I* can provide a maximum communication bandwidth that is denoted with b(I), This is available to all neighbours that can use *I*. Figure 1 shows a network where smartphones, laptops, tablets and mobile phones can communicate point-to point by using various protocols and interfaces such as UMTS, Satellite, 4G, GSM, Bluetooth, LTE, Edge, WiFi and IRdA.

All edges, that are all connections, can be established by using at least an interface. It is worth mentioning that some devices could not be directly connected although they have common interfaces. This can be a consequence of various reasons such as pathloss [12] (e.g., the presence of obstacles or long distances) and the protocol that is used. A full instance can be defined by providing the cost of each device to switch a certain interface on and the related bandwidth. This is a feature that is strictly related to the interface, unlike the energy that is spent by each device to switch a specific interface on. This cost can vary a lot when different devices are considered. We use a simplified model where the cost is referred to the battery percentage that is consumed by each device. This is the same for each device when the same interface is considered amongst the entire network. Different assumptions can lead to different problems that highlight other aspects of the interconnection.

In the last decade, multi-interface networks have been widely investigated in different contexts, especially by focusing on the advantages of using the various interfaces of each device. In this setting, various well-known network optimisation problems have been reconsidered [13,14]. In particular, network connectivity [15,16] and routing [17] have been extensively studied. Combinatorial problems of multi-interface networks have been also extensively explored in Caporuscio et al. [18]. This paper, as well as [19] and [20,21,22,23], investigate different facets of one of the most challenging problems in the context of multi-interface networks, that is the so called *Coverage* problem. It consists in finding the cheapest way to establish all the connections defined by an input graph *G*, no matter the interface used to accomplish each connection. The problem only asks to ensure that for each edge of *G*, there is a common active interface at its endpoints. The objective is to minimise the overall cost of activation in the network. Another interesting objective function that has been studied concerns the minimisation of the maximum cost on a single node [24].

In Athanassopoulos et al. [25] as well as in Kosowski et al. [26], the authors study the multi-interface networks *Connectivity* problem. This aim at finding the cheapest way to provide network connectivity. More precisely, the Connectivity problem requires to activate a set of interfaces at each node in order to ensure a path that connects every couple of nodes that belongs to the network. The cost of the interfaces activation must be minimised. In D’Angelo et al. [24], the authors take a completely different approach where the main goal is the minimisation of maximum cost of a single node.

The Connectivity problem is a generalisation of the Minimum Spanning Tree problem. It is worth mentioning that the costs for Connectivity are not on the edges but on the nodes’ interfaces. The same interface can be used by a node to establish different connections in order to save energy. This uncovers the advantages and the higher complexity of the multi-interface network Connectivity problem.

In Kosowski et al. [26], the authors study the *Cheapest path* problem. This has the main goal of finding a set of interfaces that must be activated in order to allow communication between two nodes. The set of interfaces that are activated must be the least cost in terms of activation energy. This is a generalisation of the well-known shortest path problem between two nodes. It is worth mentioning that this problem maintains a polynomial time computational complexity when solved in the context of the multi-interface networks.

In Kosowski et al. [27], the authors study the problem of the *Maximum Matching*. This finds a maximum subset of connections that can be established concurrently without sharing any common nodes. More precisely, a solution is composed by a set of disjoint edges of the network graph, thus, a node appears in the solution no more than once. This problem is rather complex in the context of the multi-interface networks. In fact, a simplified instance of the *Maximum Matching* problem where the network graph *G* has no costs and bandwidths is already very difficult to solve. In fact, when a solution connects two edges by using the same interface, these edges cannot be directly connected by a third one as otherwise this would result as established since both its endpoints share the same active interface. This then invalidates the solution.

In D’Angelo et al. [28] and in Audrito et al. [29], the authors study the bandwidth constraint problem. This concerns flow on multi-interface networks. In these papers, each interface is associated with a specific bandwidth. The *Maximum Flow* and *Minimum Cost Flow* problems aim at finding a connection between two nodes by considering the bandwidth constraints. The Maximum Flow problem aims at maximising the bandwidth between the two nodes. All the network interfaces are assumed to be active. In this way all the permitted connections can be established. In this context, a flow function that guarantees the maximum communication bandwidth between the two nodes has been studied. This problem is a generalisation of the Maximum Flow problem for standard networks. The Minimum Cost Flow problem aims at finding a communication sub-network between two nodes that has a minimum energy consumption cost and ensures at least a given communication bandwidth *B*. More precisely, the problem finds the set of active interfaces that has minimum cost and ensures that a certain node *s* can exchange data with another node *t* with a bandwidth not smaller than *B*. Although the solution can be a path between *s* and *t*, a more complex graph can be required. This has nodes with active interfaces that depend on the network topology. This problem is a generalisation of the Minimum Cost Flow problem for standard networks.

## 3. Experimental Setup

In this section, we explain all details concerning the settings established in our experiments. In particular, we provide hardware, software and strategies chosen along the motivations that led to such choices.

### 3.1. Hardware and Circuit Setting

In order to measure the energy consumed by a smartphone during our file transferring operations, we need to choose various settings and external devices to be used. Clearly, there might be many factors influencing the battery drain. Since our goal is to measure the consumption due only to the file transferring, we need to consider the lightest environment for the smartphone, hence excluding the consumption due to underlying apps. We cannot rely on third party apps or built-in battery monitoring as it is never clear the level of reliability of such measurements, including the cost of running the monitoring itself. To this respect we follow a similar approach to [3], where the smartphone is connected with external devices. In order to be accurate, we have used very precise potentiostats. These provide a constant power supply to the connected smartphone and allow the recording of energy consumption with very high accuracy.

**Figure 2:**
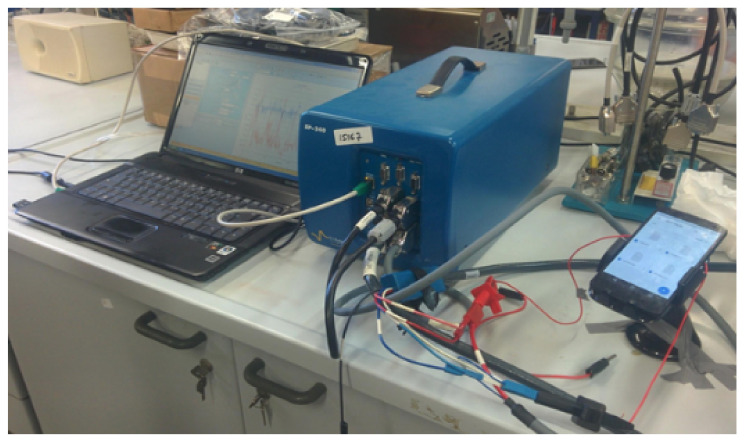
Experiments in our electrochemistry laboratory.

Table 1 shows all smartphones that have been used in our experiments. Smartphones have been selected in order to cover various Android releases that range from 6.0.1 to 8.1. The aim is to understand the dependability of the experiments with respect to the operating system. Also, we make sure to cover a wide range of interfaces that are WiFi at 2.4 and 5 GHz, various Bluetooth versions and the 4G cellular connection. Further reasons for our choices concern the availability of some smartphones along with economic constraints. In Figure 2 we show the set up of the devices for running our experiments.

It is worth mentioning that many smartphones cannot operate without the battery due to control chips installed. Without the equipment available in the laboratory we should have excluded some smartphones from our studies. Moreover, the accuracy provided with the used equipment is much higher than that for instance achieved by the simple circuit used in [3] where a smartphone is connected to an oscilloscope through a resistor.

**Figure 3:**
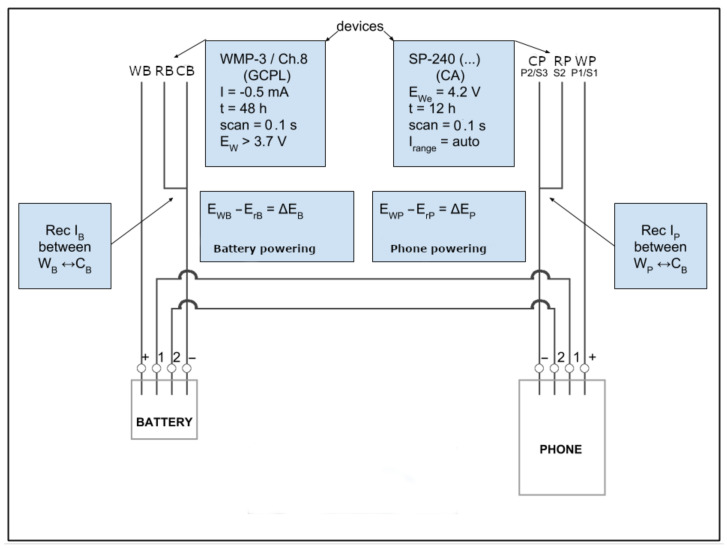
Circuit schema used to collect time and energy consumption data.

**Figure 4:**
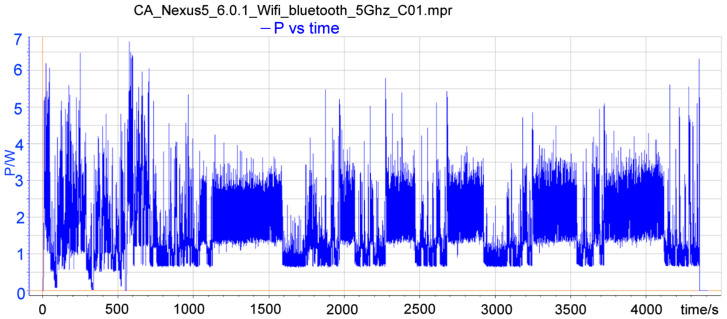
Plot of the energy consumption required for a file transfer from a smartphone LG Nexus 5 by means of the concurrent usage of interfaces WiFI and Bluetooth.

Figure 3 shows the circuit we have used for our experiments on each smartphone. We have two potentiostats that are WMP-3 (https://www.bio-logic.net/products/potentiostat-galvanostat-eis/vmp3-modular-16-channels-potentiostat/) and SP-240 (https://www.bio-logic.net/products/potentiostat-galvanostat-eis/sp-240-4-research-grade-potentiostatgalvanostatfra/). A potentiostat is an electronic device that can be used to keep the voltage difference between a working electrode and a reference electrode constant, while circulating and measuring a current flow between the working and the counter electrodes. Thus, the controlled variable is the cell potential and the measured variable is the cell current. The SP-240 potentiostat is connected to the smartphone. The SP-240 device ensures the potential difference $E_{p}$ (i.e., $E_{p}=E_{WP}-E_{rP}$) between the potential $E_{WP}$ of the working electrode (WP) and the potential $E_{rP}$ of the reference electrode (RP) is kept constant at $E_{We}=4.2$ V. The current $I_{p}$ circulating between the working (WP) and the counter (CP) electrodes is monitored every 0.01 ms in order to plot its value over the time. An example of the output obtained during an experiment of file transfer from a smartphone LG Nexus 5 by means of the concurrent usage of interfaces WiFI and Bluetooth can be seen in Figure 4.

This allows us to calculate the energy consumption in Wh (Watt times hour). More precisely, we use the following formula in order to calculate the energy consumption of an experiment:

$$\int_{t_{1}}^{t_{2}}E_{p}I_{t}dt$$

where $t_{1}$ and $t_{2}$ are the times at which the experiments started and ended, while $I_{t}$ is the current consumed at time dt by the smartphone. The time t=12 h inside the SP-240 box of Figure 3 shows the duration of the experiments.

While the use of the bare potentiostat SP-240 is sufficient for the Nokia N1 and the Samsung S4, this setup is not suitable for the LG Nexus 5 and the Motorola G4. These devices require the battery to be connected to the smartphone otherwise they do not turn on. In fact, while their batteries necessarily require just two pins (i.e., + and −) for powering the smartphone, two additional pins (1 and 2) must be connected as well. Pin 1 can be a BSI (Battery Status Indicator) and is used to identify the kind of battery being used, its capacity and for battery detection. An additional pin 2 can represent an internal temperature sensor as variable resistor which is useful as a safety signal.

We have thus connected the battery of the smartphone by using a second potentiostat VMP-3 and adding the battery of the smartphone to the circuit. This is shown in the left-hand side of Figure 3. The control pins of the battery (i.e., pins 1 and 2) are connected to the related pins of the smartphone. The battery is fully charged and it is connected to the VMP-3 potentiostat. The VMP-3 device ensures the potential difference $E_{B}$ (i.e., $E_{B}=E_{WB}-E_{rB}$) between the potential $E_{WB}$ of the working electrode (WB) and the potential of the reference electrode (RB) is kept constant at 4.5 V, which is the potential value needed for fully charging the battery, and is a value higher than the 3.7 Volts needed for turning on the smartphone. The VMP-3 potentiostat reads between the electrodes (WB) and (CB) a relatively low current of the order of −0.5 mA, close to the low current detection limit, confirming that the potentiostat is giving current to a battery which is close to its fully charged state and requires only minimal amounts of energy for monitoring of internal parameters.

### 3.2. App Implementation and Setting

We developed a Fast mUltiple inteRface transmIssion (FURI) Android app which allows the transferring of files between a sender and a receiver. These can include smartphones, tablets and pc. Sender and receiver can communicate directly, or via an access point, or by using a centralised cloud service – a beta release of our app can be found at https://github.com/Alepacox/-PublicAndroid-MultiInterface_Sender.

**Figure 5:**
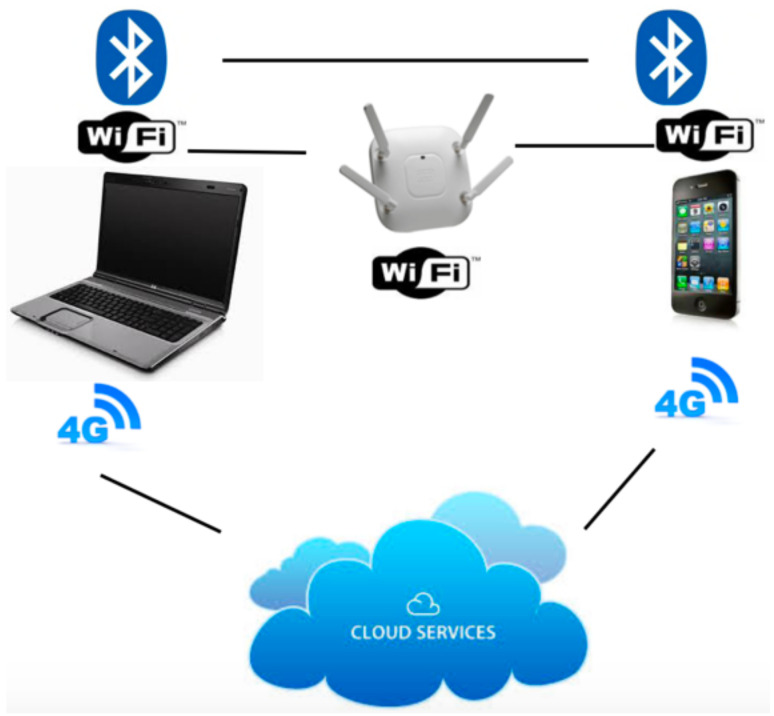
Bluetooth, WiFi and 4G architecture.

Figure 5 shows the scenario we consider in our experiments for the connections of two devices, a laptop and a smartphone, that can communicate directly via Bluetooth, by means of an access point for exploiting the WiFi connection, and by means of the 4G connection via a centralised cloud service, i.e., the network operator. Concerning the WiFi, in our experiments we have used a Cisco 2.4/5 GHz access point, whereas for the 4G connection we operated via TIM.

**Figure 6:**
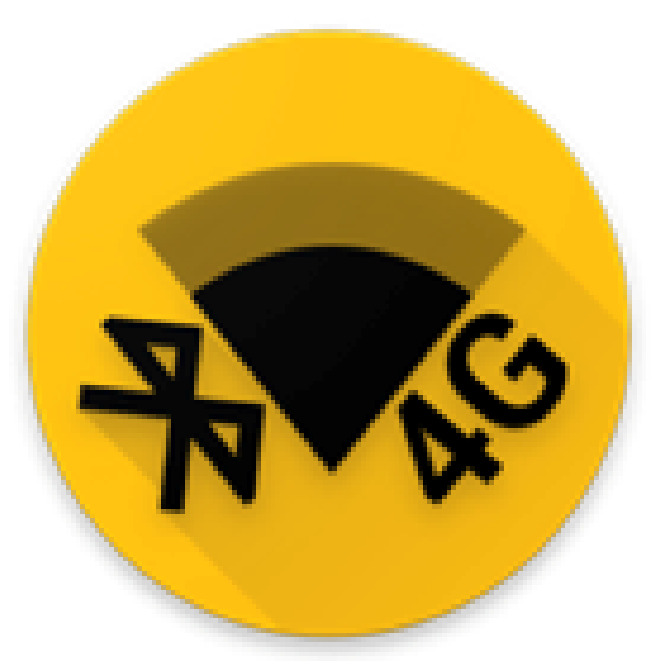
Icon of the implemented app FURI.

The use of our application (whose icon in shown in Figure 6) requires four steps to be performed: (i) communication interface selection; (ii) transferring information; (iii) transferring mode selection; (iv) file transfer.

**Figure 7:**
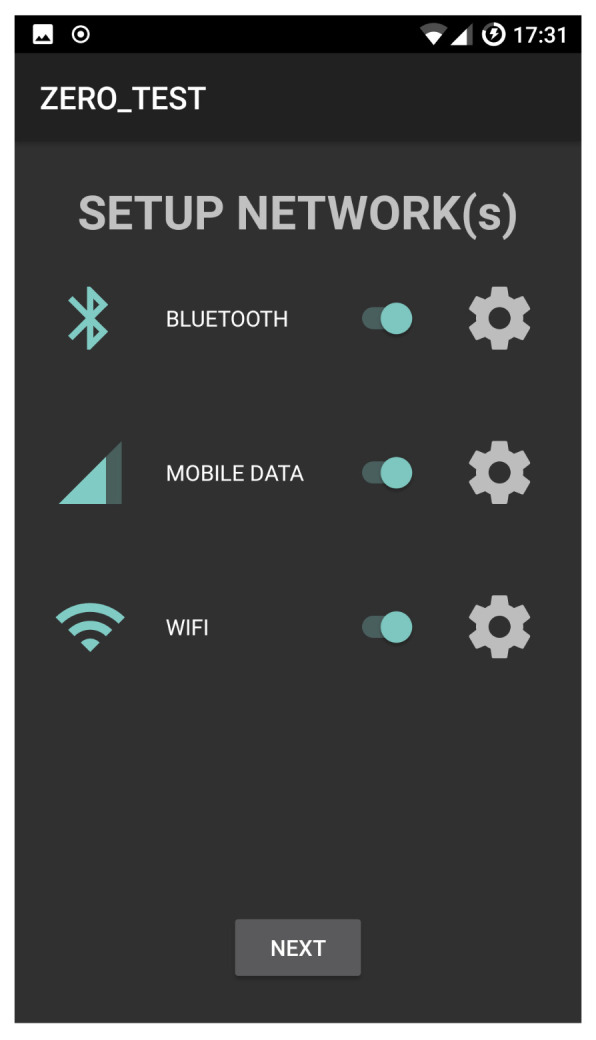
Communication interface selection panel.

Figure 7 shows the communication interface selection panel in the case that all the available interfaces are selected for transmission.

**Figure 8:**
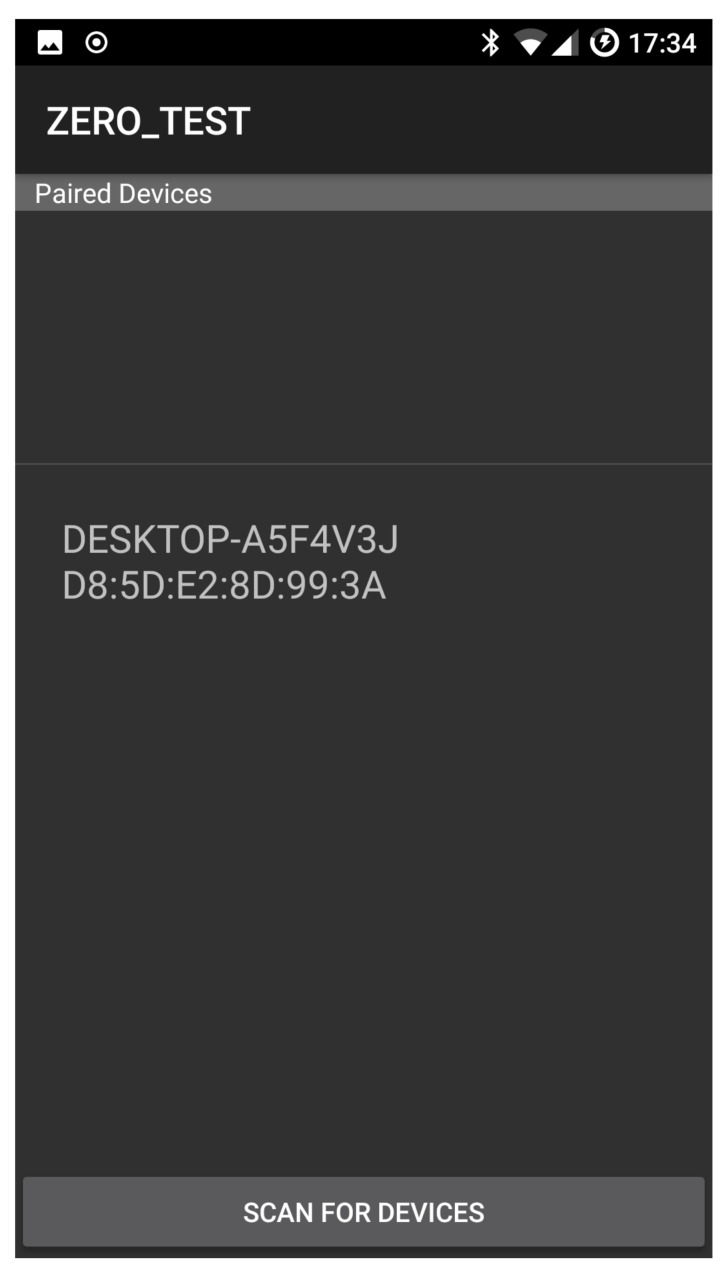
Device selection.

**Figure 9:**
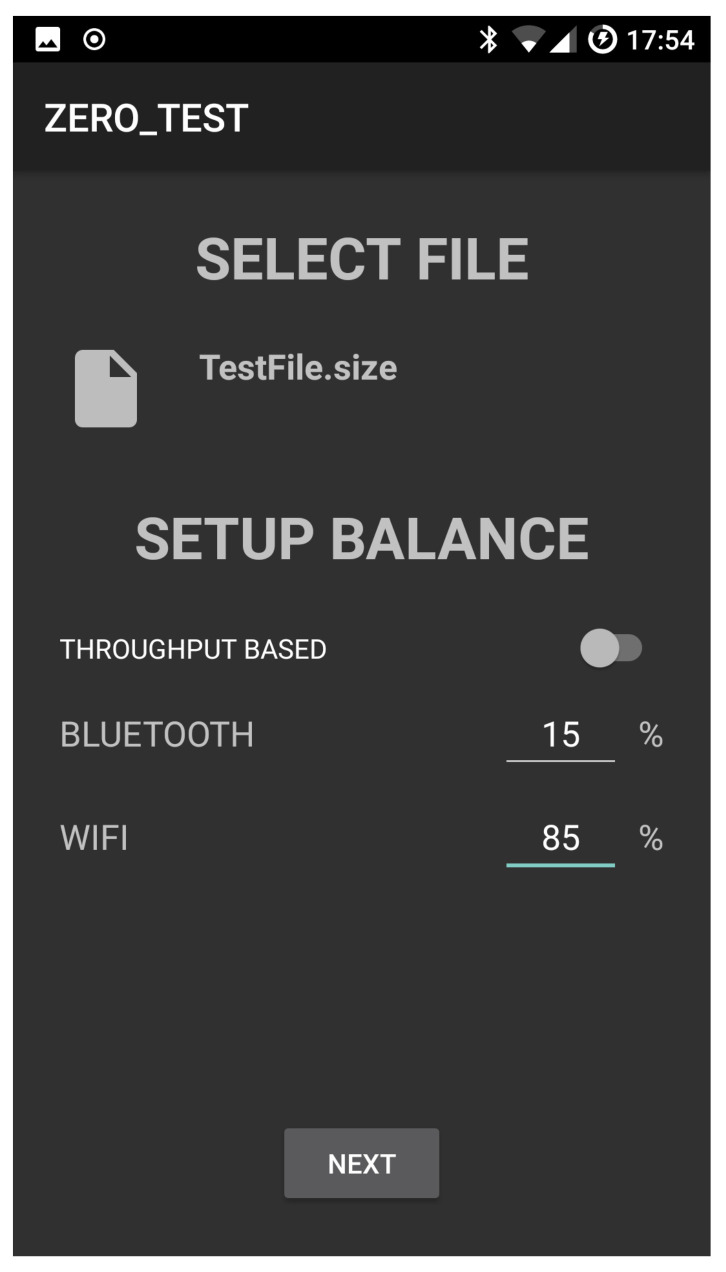
File upload and balance setup.

Figure 8 shows the device selection panel. The user can scan for devices that are connected to the same local area network or look up in a centralised directory for on-line devices. The device selection is followed by the file and the setup balance selection. Figure 9 shows the selection of the file *TestFile* and a manual setup balance of 15% Bluetooth and 85% WiFi. This means that 15% of the file will be transferred by using Bluetooth while the remaining 85% by using WiFi. The app also allows the selection of a throughput based transferring mode that optimises the transferring according to the available options and the current status of the surrounding environment. In what follows we refer to this option as *AUTO mode*. This is described in the next section.

**Figure 10:**
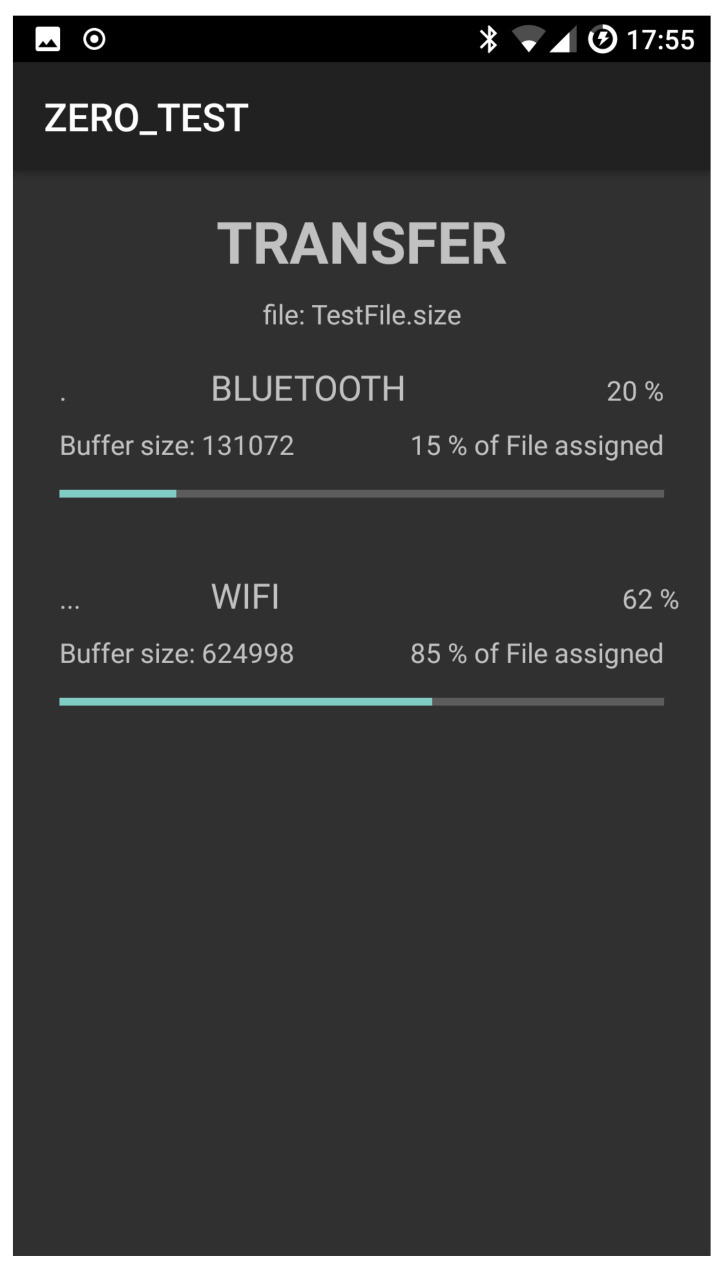
Transferring of data combining Bluetooth and WiFi.

**Figure 11:**
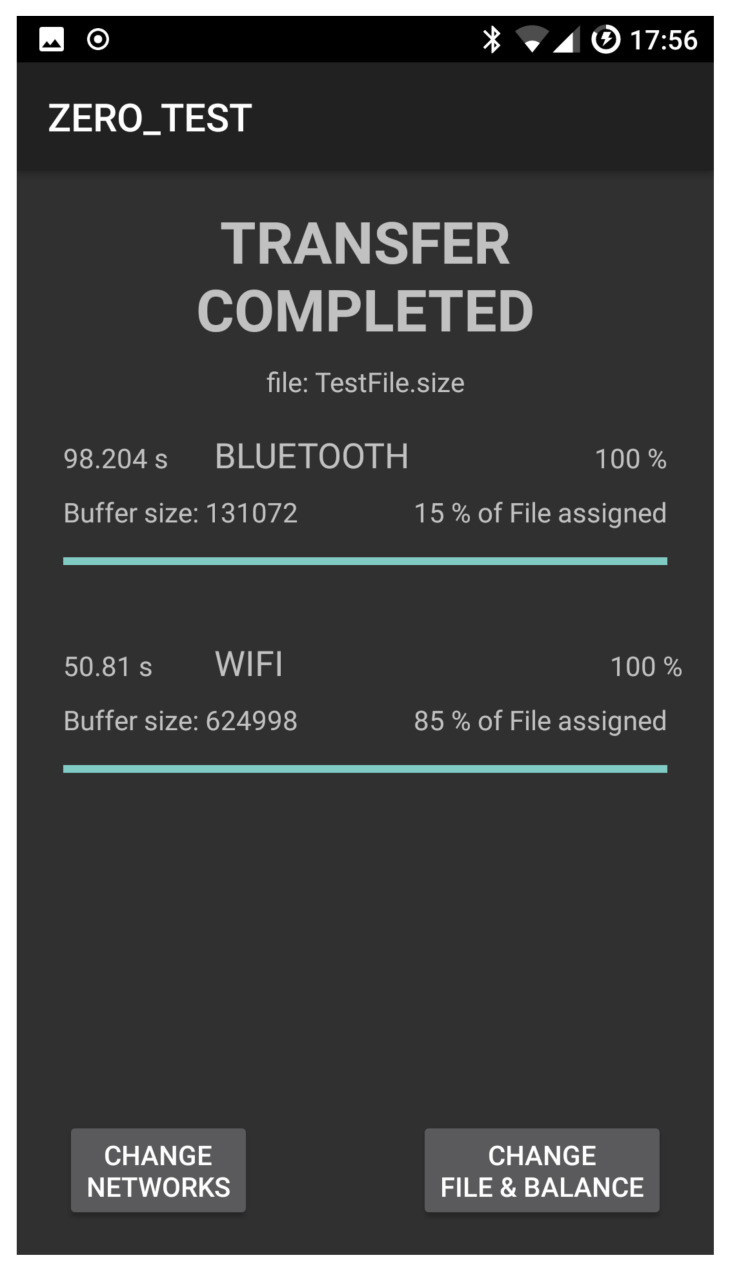
File upload and balance setup.

Figure 10 and Figure 11 show the snapshot while a file transfer is progressing and then completed, respectively. Figure 11 also contains the times spent to transfer the file to the recipient device (a TCP connection is used to transfer the file). In our experiments, the recipient device is a notebook Hp ENVY 15-j040el.

### 3.3. AUTO Mode

The AUTO mode divides the file to be transferred into different parts and allows the selected communication interfaces (e.g., Bluetooth and WiFi in our example) to transfer available parts by using their maximum transmission rate. As we are going to see in the experimental results section, manual setup balance is useful to interpret the results that are obtained by using the AUTO mode.

In what follows data[] denotes the array of bytes that must be sent. *I* denotes the set of all interfaces and $\left\{I_{1},\ldots ,I_{n}\right\}$ are elements in *I*. We denote with $T_{I_{i}}$ the throughput of the interface $I_{i}$ and with $T=\{T_{i_{1}},\ldots ,T_{i_{n}}\}$ the set of all interfaces’ throughputs. The array data[] can be split into different subarrays that can be sent over different iterations {1,…,m} with m≥1. A data percentage parameter α, with 0<α≤1, can be used to divide the array data[] into $\alpha^{-1}$ subarrays with size G=sizeof(data)×α bytes each. We use $d_{k}$ to denote the subarray data[k − 1,k + G − 1] that is sent during the iteration *k*. The data $d_{k}$ is further split into different parts $\left\{d_{k,I_{1}},\ldots ,d_{k,I_{n}}\right\}$ and each part $d_{k,I_{j}}$ is sent by using the interface $I_{j}$.

Algorithm 1 shows the pseudo-code of the AUTO mode. The basic idea is to estimate the throughput of each interface in various iterations. This is used in order to split the data into different parts. Each part is sent by using a different interface. The best performance in terms of transferring time can be achieved when all the interfaces end the transmission of the data at the same time.
**Algorithm 1** Pseudo-code AUTO mode
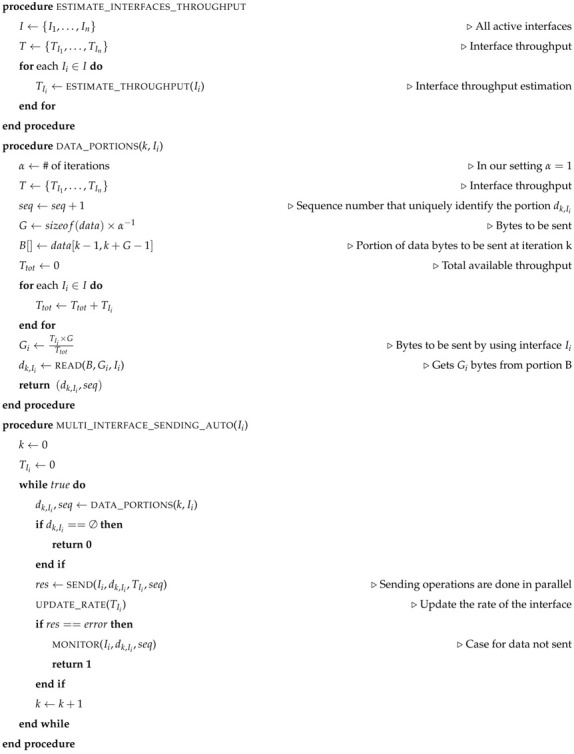


The estimate_interfaces_throughput procedure of Algorithm 1 is used at the beginning to estimate the throughput of each interface $I_{i}$. This is stored in the variable $T_{i}$. The procedure data_portions$\left(k,I_{i}\right)$ is used in order to get the portion $d_{k,I_{i}}$ of bytes that must be sent via the interface $I_{i}$. The procedure calculates the amount of bytes to be sent during the iteration *k* and store it in a variable *G*. The array B[] is used to store the subarray of data[] that needs to be sent. Only $G_{i}$ bytes of B[] are sent by using the interface $I_{i}$. The amount $G_{i}$ is proportional to the throughput that the interface $I_{i}$ has with respect to the total one (i.e., the sum of the throughput of all interfaces). The procedure read is used to get the $G_{i}$ bytes from B[] that must bu sent by using the interface $I_{i}$. A unique sequence number seq is used in order to rebuild the data at the recipient side.

The multi_interface_sending_auto$\left(I_{i},e\right)$ procedure shows the sending of data[] by using the interface $I_{i}$. The procedure data_portions is used to obtain the array of bytes $d_{k,I_{i}}$. This contains the portion of data[] that the interface $I_{i}$ needs to send during the iteration *k*. When $d_{k,I_{i}}$ is empty all data have been sent and the process terminates with success. Data are sent by using a reliable send which also updates the throughput $T_{i}$. The update_rate$\left(T_{I_{i}}\right)$ procedure is used to update the array of throughput *T*. When the data cannot be sent (e.g., the interface has been switched off) the process uses a monitor$\left(I_{i},d_{k,I_{i}},seq\right)$ to notify that the data $d_{k,I_{i}}$ has not been sent. This can be sent by using another interface (if any) or the entire file transfer process can be aborted.

In our experiments, we have used α=1 that is the data were sent by using a single iteration. In fact, the interface throughput was very stable since we have performed our experiments in the best case scenario where the WiFi was delivered via a dedicated access point that was in Line of Sight with the sender. There was no interference caused by other surrounding WiFi. The 4G main antenna was in Line of Sight 500 m away from the cell phone. We also noticed that the throughput was greatly improved by dividing each part of data $d_{k,I_{i}}$ (to be sent by the interface $T_{i}$ at iteration *k*) into smaller chunks $\left\{p_{1},\ldots ,p_{n}\right\}$. We added to each $p_{i}$ the header needed to rebuild the message at the recipient side. The size of the part $p_{i}$ was chosen as large as possible to increase the throughout, but also small enough to avoid fragmentation by the underlying layers.

At an implementation level, for each interface $I_{i}$ we run a different process which executes the procedure multi_interface_sending_auto$\left(I_{i}\right)$. A main process is responsible for creating the interface processes and running the procedures data_portions, monitor and update_rate. A failure on one of the interface multi_interface_sending_auto procedure makes the entire sending to stop. We have also investigated the use of more iterations for sending files. This does not give any advantage in the studied environment in terms of transferring time since our connections are quite stable in terms of throughput. However, our implementation is ready to approach real case scenarios where connections are less stable. In that case, a main issue would be that to obtain accurate energy estimations as we have done in our laboratory.

### 3.4. Android Peculiar Settings

Our main goal is to obtain the measure of the time and the energy spent as consequence of a file transfer, thus we need to exclude any other factors influencing the battery drain. To this end, we have considered clean installations of Android operating systems where no additional apps have been installed. We have excluded background processes that can start during the execution of the experiments and monitor those processes whose execution cannot be excluded. For instance we have not prevented a 4G phone call while the 4G is used to run our experiments. In our app, each communicating interface (i.e., Bluetooth, WiFi and 4G) is implemented with a different process. A peculiar setting which may greatly affect the experiments is the scheduling policy of the Android operating systems. More precisely, Android may suspend the execution of the communicating interface processes. This can cause the interface to drain energy without delivering any data. We have solved this problem by setting the priority of the communicating interface processes as high as Android kernel ones.

We make sure that the energy consumption is stable at the sender side then we run our file transfer app and measure the increase in energy consumption. We do not consider the costs to switch on the interfaces since we assume they are already on. In fact, users rarely turn 4G data, WiFi and Bluetooth off. Moreover, switching off those interfaces does not actually turn them off (they will continue to work, see e.g.,  https://www.independent.co.uk/life-style/gadgets-and-tech/news/ios-11-apple-iphone-ipad-bluetooth-wifi-switching-off-turn-toggle-control-center-a7959301.html). This is how the operating system ensures the correct working of various basic services. We have also verified that turning off and on interfaces does not have an evident impact on the energy consumption.

Finally, we have also considered that different smartphones can take different times to read the file from the local memory. To this respect, we have installed different Android versions on the same smartphone. This allows us to check the energy consumption of different Androids, nonetheless we have considered different smartphones by considering the speed access to the local smartphone memory.

## 4. Experimental Results

In this section, we show the experimental results experienced during our studies on file transfers from smartphones. In particular, we focus on two main measurements that are time and energy consumption required to perform the transfers. To summarise, the interfaces explored are Bluetooth, WiFi at 2.4 GHz, WiFi at 5 GHz, and 4G. We test file transfers either by means of stand-alone interfaces or coupling some interfaces. In particular, our main investigation focuses on couples (WiFi at 2.4 GHz, 4G), (WiFi at 5 GHz, 4G), (WiFi at 2.4 GHz, Bluetooth) and (WiFi at 5 GHz, Bluetooth). Other settings and experiments are not reported as they do not add much to the discussion. The couplings have been performed either by manually partitioning the file to transfer into fixed percentages per interface, or in what we have called the AUTO mode that optimises on performances. The files used for our transfers have been randomly generated and vary on size. We consider files of size 1 Mb, 10 Mb when considering Bluetooth, up to 100 Mb and 500 Mb when considering WiFi and 4G. In fact, performances when Bluetooth is used are already well-known to be effective only for files of small size, even smaller than 1 Mb [3]. Such a behaviour cannot certainly ameliorate in coupling Bluetooth with interfaces like WiFi and 4G that admit much better performance characteristics. However, some settings involving Bluetooth still provide some slight improvement.

### 4.1. Results for WiFi at 2.4 GHz and 4G

In this section, we show and comment on results obtained for file transfers by means of interfaces WiFi at 2.4 GHz, 4G, and their combination on a Samsung Galaxy S4 with Android 7.1.2.

**Figure 12:**
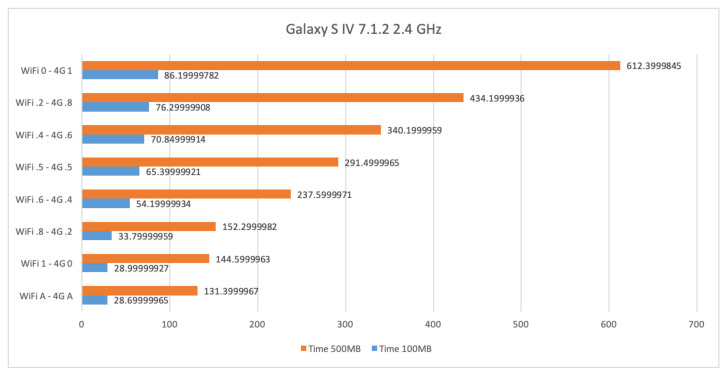
Time required for transferring files of size 100 Mb and 500 Mb by means of WiFi at 2.4 GHz and 4G on a Samsung Galaxy S4 with Android 7.1.2. Apart for the last line which refers to the AUTO mode, from top to bottom, the percentage of using WiFi goes from 0% to 100%, whereas the one for 4G is decreased from 100% to 0%. The best performance is obtained by means of the AUTO mode.

Figure 12 shows the time required for sending files of size 100 Mb and 500 Mb in various settings involving WiFi at 2.4 GHz and 4G. When only 4G is used, the time required to transfer 100 Mb is 86.2 s whereas for 500 Mb it is 612.4 s. Such results decrease to 29 and 144.6 s, respectively, when only WiFi at 2.4 GHz is used. The best result is obtained by using the AUTO setup that is 28.7 and 131.4 s, respectively. Basically, in the AUTO settings, WiFi and 4G can fully exploit their bandwidth characteristics by sending the chunks of the file at their maximum speeds; WiFi sends slightly more than the 80% of the file while 4G the remaining part. In particular, according to the interfaces throughput experienced by means of procedure estimate_interfaces_throughput shown in Algorithm 1, the precise percentages assigned to WiFi and 4G are close to 84% and 16%, respectively. Clearly, such values are sensible to the current available throughput of the interfaces. It is worth noting that the closest manual model experienced that assigns 80% to WiFi and 20% to 4G does not provide such similar performances. In fact, from the manual settings the best case seems to be the WiFi in its stand alone setting. This reveals a very interesting behaviour of our new technique for fastening the file transfer operation which comes out to be rather sensitive to different assignments of percentages. We can notice that the throughput obtained by using WiFi and 4G in the AUTO mode gets better as the size of the file increases. The reason why WiFi is in charge of a big percentage of chunks with respect to 4G can be motivated by the fact that in our experiments the WiFi connection is of peer-to-peer fashion whereas the 4G requires cloud services to be established. It follows that 4G results much slower than WiFi, hence high percentage of 4G slows down the whole transfer process. Increasing the percentage of the WiFi usage results in better performance time-wise. However, the setting induced by our AUTO mode is instead the best trade-off where not much has been assigned to 4G but still it makes the difference with respect to the stand-alone WiFi setting.

**Figure 13:**
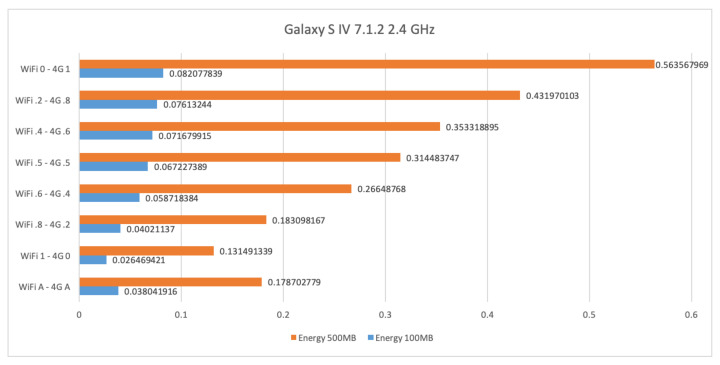
WiFi at 2.4 GHz and 4G energy consumption results for the Samsung Galaxy S4.

Figure 13 shows the energy consumption in Wh (watt times hour) for sending files of size 100 Mb and 500 Mb by using WiFi at 2.4 GHz and 4G. The energy to transfer 100 Mb and 500 Mb is about 0.08 and 0.563 Wh, respectively, when only 4G is used. The energy consumption decreases to 0.026 and 0.131 Wh, respectively, when only WiFi is used. The AUTO mode actually consumes slightly more than the stand-alone WiFi, that is 0.038 and 0.178 Wh, respectively.

In practice, the experiments show that coupling WiFi at 2.4 GHz with 4G can provide some gain in terms of time required to transfer a file at a slight increasing of the energy consumed. In particular, with respect to the transfer of 500 Mb we gain 13.2 s at the cost of roughly 0.047 Wh. Moreover, as the size of the file to transfer increases, the time gained fast increases, whereas the gap about the energy spent becomes negligible. In fact, concerning the results obtained with respect to the transfer of 100 Mb, the gain of the AUTO mode with respect to the stand-alone Wifi is just 0.3 s, whereas the extra energy spent is 0.011. Such numbers can become really effective in contexts where the time is crucial because the energy might be spent also for other operations meanwhile. To this respect, we imagine that a suitable application might be constituted by a drone that needs to collect data from some base station while flying. In such a scenario, making the transferring operation shorter in time leads to save energy for the hovering, whereas the extra energy spent for the file transfer becomes negligible.

### 4.2. Results for WiFi at 5 GHz and 4G

In this section, we show and comment on results obtained for file transfers by means of interfaces WiFi at 5 GHz, 4G, and their combination on a Samsung Galaxy S4 with Android 7.1.2.

**Figure 14:**
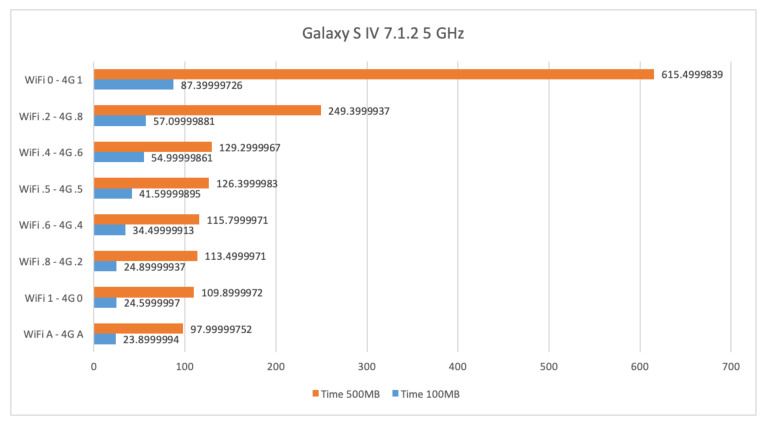
Time required for transferring files of size 100 Mb and 500 Mb by means of WiFi at 5 GHz and 4G on a Samsung Galaxy S4.

Figure 14 shows the time required for sending files of size 100 Mb and 500 Mb by using WiFi at 5 GHz and 4G. The time to transfer 100 Mb and 500 Mb is 87.4 and 615.5 s, respectively, when only 4G is used. Such results decrease to 24.6 and 109.9 s, respectively, when only WiFi is used. The best result is obtained by using the AUTO mode that is 23.9 and 98 s, respectively. Similarly to the case involving the WiFi at 2.4 GHz, in the AUTO setting WiFi and 4G send chunks of file at their maximum speed when WiFi sends slightly more than the 80% of the file. Similar outcomes can also be inferred. The obtained partitioning is consistent with the characteristics of the involved interfaces. Again, we notice that the throughput obtained by using WiFi and 4G in AUTO mode gets better as the size of the file to transfer increases.

In general, the usage of WiFi at 5 GHz leads to better throughput performance with respect to those obtained with the WiFi at 2.4. This is due to two main reasons: (i) Wifi at 5 GHz is faster than the 2.4 GHz one; and (ii) Wifi at 2.4 GHz suffers of interference problem. In fact, the 2.4 GHz frequency is used by many devices (e.g., wireless mouses and keyboard) and by other technologies such as Bluetooth and ZigBee.

**Figure 15:**
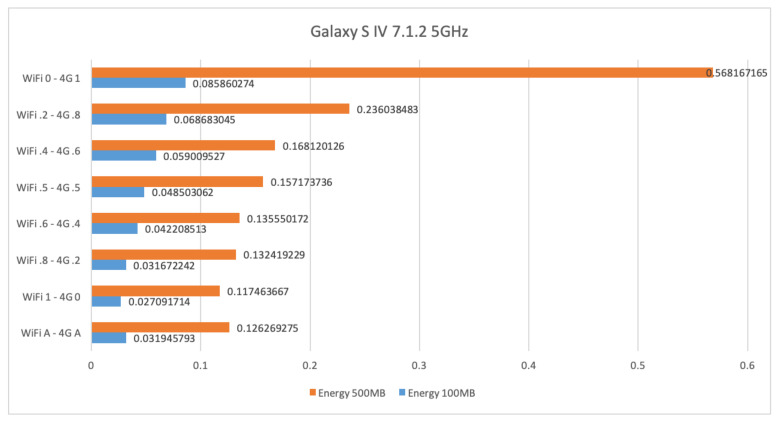
WiFi at 5 GHz and 4G energy consumption results for the Samsung Galaxy S4.

Figure 15 shows the energy consumption in Wh for sending files of size 100 Mb and 500 Mb by using WiFi at 5 GHz and 4G. The energy to transfer 100 Mb and 500 Mb is roughly 0.085 and 0.568 Wh, respectively, when only 4G is used. This energy consumption decreases to 0.027 and 0.117 Wh, respectively, when only WiFi is used. The energy consumption when using the AUTO mode is instead of 0.032 and 0.126 Wh, respectively. These are slightly worse than the WiFi results. However, the throughput gets better as the size of the file increases.

### 4.3. Results for WiFi at 2.4 GHz and Bluetooth

In this section, we show and comment on results obtained for file transfers by means of interfaces WiFi at 2.4 GHz, Bluetooth, and their combination on different smartphones and operating systems.

**Figure 16:**
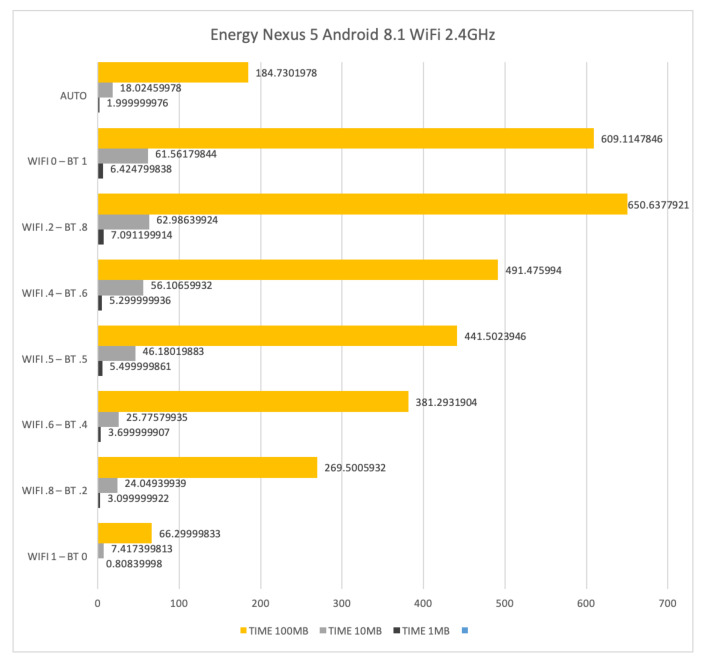
Time required for transferring files of size 1 Mb, 10 Mb and 100 Mb by means of WiFi at 2.4 GHz and Bluetooth on a LG Nexus 5 with Android 8.1.

Figure 16 shows the time required for sending files of size 1 Mb, 10 Mb and 100 Mb by using WiFi at 2.4 GHz and Bluetooth on the LG Nexus 5. The time to transfer 1 Mb, 10 Mb and 100 Mb is 6.42, 61.56 and 609.11 s, respectively, when only Bluetooth is used. Such results decrease to 0.80, 7.413 and 66.3 s, respectively, when only WiFi is used. The AUTO mode requires more than the double of the time spent by the stand-alone WiFi setup. This confirms the bad behaviour of WiFi at 2.4 GHz concerning interference.

**Figure 17:**
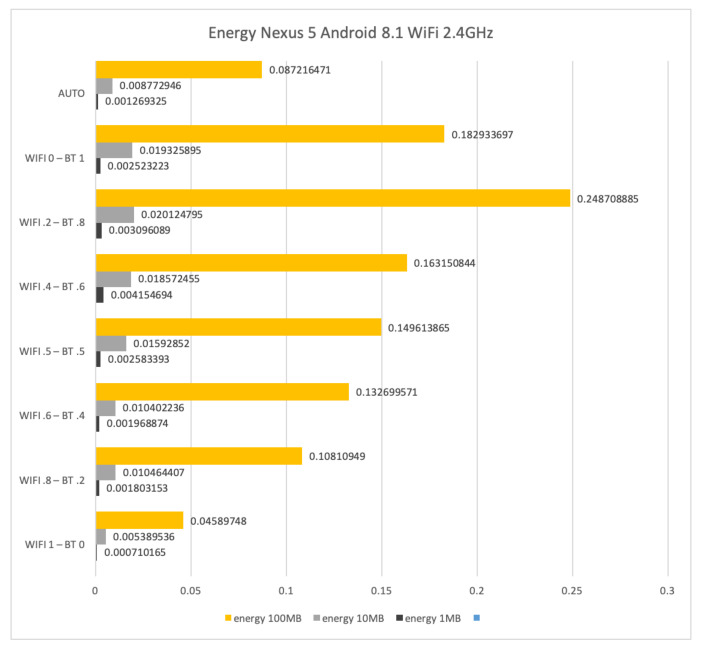
WiFi at 2.4 GHz and Bluetooth energy consumption results for the LG Nexus 5 with Android 8.1.

Figure 17 shows the energy consumption in Wh for sending files of size 1 Mb, 10 Mb and 100 Mb by using WiFi at 2.4 GHz and Bluetooth on the LG Nexus 5. The energy to transfer 1 Mb, 10 Mb and 100 Mb is 0.0025, 0.0193 and 0.1829 Wh, respectively, when only Bluetooth is used. Such results decrease to 0.0007, 0.0054 and 0.0459 Wh, respectively, when only WiFi is used. Again, the AUTO mode consumes more than the stand-alone WiFi setup.

**Figure 18:**
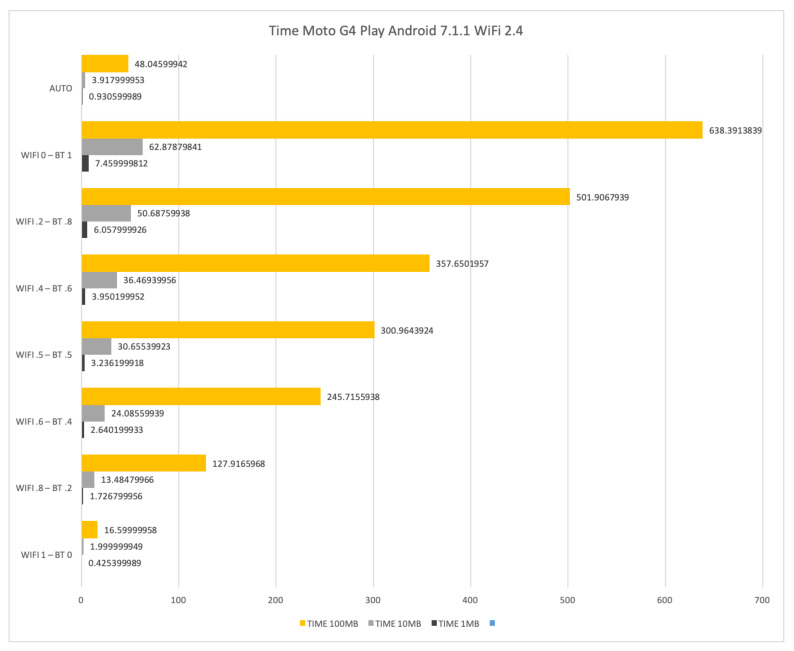
Time required for transferring files of size 1 Mb, 10 Mb and 100 Mb by means of WiFi at 2.4 GHz and Bluetooth on a Motorola G4 Play.

**Figure 19:**
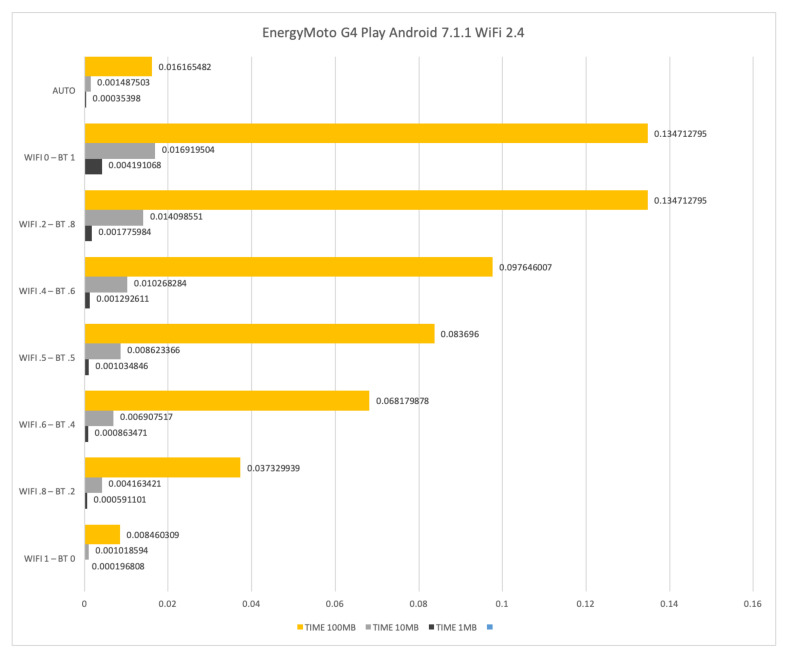
Time required for transferring files of size 1 Mb, 10 Mb and 100 Mb by means of WiFi at 2.4 GHz and Bluetooth on a Motorola G4 Play.

Similar negative results in terms of both time and energy consumption have been experienced on a Motorola G4 Play with Android 7.1.1, see Figure 18 and Figure 19. For that reason we do not report results about NOKIA N1 where only WiFi at 2.4 GHz is available.

### 4.4. Results for WiFi at 5 GHz and Bluetooth

In this section, we show and comment on results obtained for file transfers by means of interfaces WiFi at 5 GHz, Bluetooth, and their combination on the LG Nexus 5 with Android 8.1.

When WiFi at 5 GHz is coupled with Bluetooth, it is possible to obtain better results with respect to the usage of WiFi at 2.4 GHz. However, differences between the coupled interfaces with respect to their stand-alone usage do not provide significant improvements. Moreover, the best performances have been obtained with the latest version of Android we could test, that is version 8.1.

**Figure 20:**
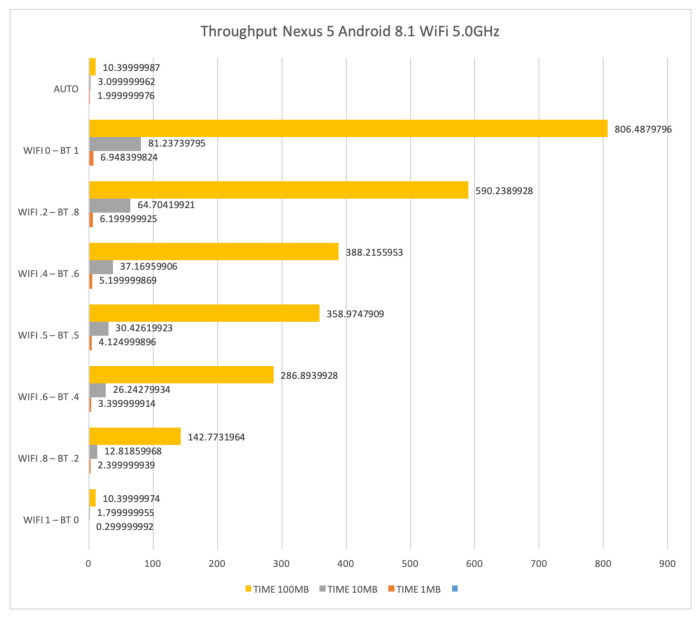
Time required for transferring files of size 1 Mb, 10 Mb and 100 Mb by means of WiFi at 5 GHz and Bluetooth on a LG Nexus 5.

**Figure 21:**
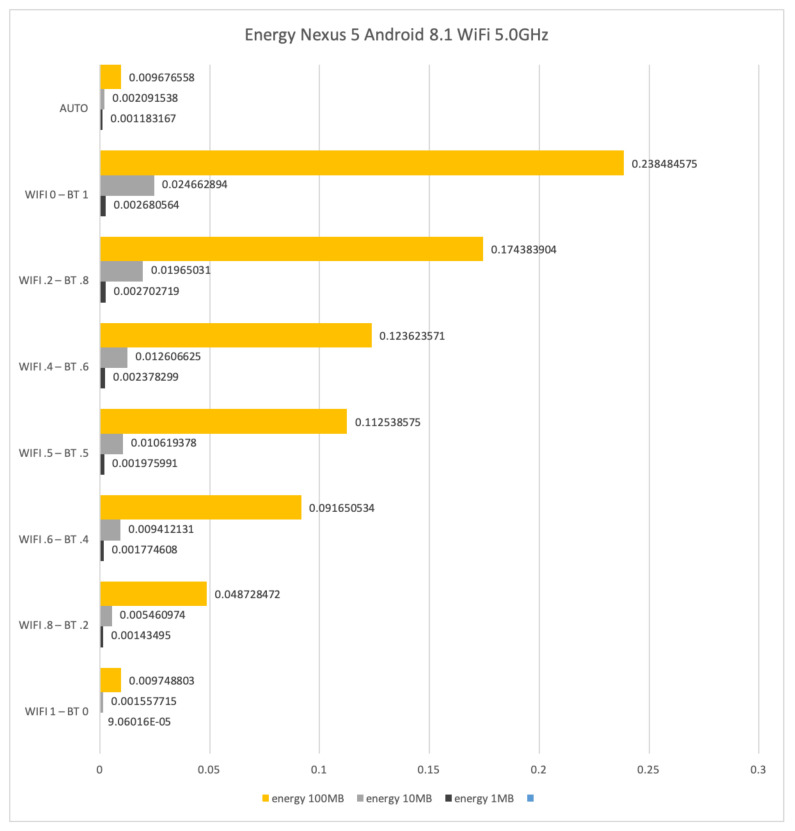
Time required for transferring files of size 1 Mb, 10 Mb and 100 Mb by means of WiFi at 5 GHz and Bluetooth on a LG Nexus 5.

Figure 20 and Figure 21 show the obtained results on the LG Nexus 5 with Android 8.1.

Figure 20 shows the time required for sending files of size 1 Mb, 10 Mb and 100 Mb by using WiFi at 5 GHz and Bluetooth. The time to transfer 1 Mb, 10 Mb and 100 Mb is 6.42, 61.56 and 609.11 s, respectively, when only Bluetooth is used. Such results decrease to 0.81, 7.41 and 66.3 s, respectively, when only WiFi is used. The AUTO mode requires basically the same amount of time than the stand-alone WiFi setup for the 100 Mb file, whereas there is still some loss for files of 1 Mb and 10 Mb. This is somehow counter-intuitive as the low throughput of the Bluetooth is expected to be effective only for files of very small dimensions.

Figure 21 shows the energy consumption in Wh for sending files of size 1 Mb, 10 Mb and 100 Mb by using WiFi at 5 GHz and Bluetooth. The energy to transfer 1 Mb, 10 Mb and 100 Mb is 0.0025, 0.0193 and 0.1829 Wh, respectively, when only Bluetooth is used. Such results decrease to 0.0007, 0.0053 and 0.0459 Wh, respectively, when only WiFi is used. Similar considerations obtained for the above results on time about the AUTO mode stand also here.

Apart for the considerations already discussed about WiFi at 2.4 or 5 GHz, the results with Android 8.1 show a better behaviour with respect to those on previous versions of the operating system when the coupling strategy involves WiFi with Bluetooth. This is possibly due to the increasing attention of developers to energy constrains.

## 5. Concluding Remarks

In this paper, we have considered the very basic task of file transfer from a smartphone for backup purposes. The transferring was conducted by testing all the available interfaces in their stand-alone setting as well as by concurrent connections. When more than one interface is used for the file transfer operation, the file is divided into chunks that are sent toward the selected interfaces at different rates. By means of extended experiments we have seen how time can be saved when coupling WiFi at 5 GHz with 4G connections. Whereas, there are no particular coupling settings that provide useful gain in terms of energy consumption. This trend was somehow expected, even though the extra energy seems to become negligible as the size of the file grows. Bluetooth is confirmed to be useful in its stand-alone usage for files of very small sizes. When coupled with another interface, it seems not to provide particular improvements. The best results were obtained with Android 8.1 where it seems energy is managed in a better way with respect to preceding operating systems. However, the results on Bluetooth are clearly due to the big gap between its maximum throughput and those provided by 4G or WiFi. In this respect, we can expect much better behaviours by coupling WiFi at 5 GHz with 5G as soon as this last technology becomes easily available. The negligible improvements obtained with Bluetooth are basically the main reason why we did not report further experiments with Bluetooth, WiFi and 4G all concurrently connected. Actually, we had some preliminary results to this respect but the outcome seems to be even worst than expected as the managing of all such interfaces by means of the operating system was really costly in terms of energy.

With our experiments, we have answered to a long term question about the effectiveness of multi-interface networks with respect to the minimisation of both time and energy consumption. In order to accomplish our experiments, we had to implement an ad-hoc Android app, facing various technical details. The app is very light and can be potentially installed on any compatible device, hence opening a wide range of applications. We also wanted to conduct experiments on an iPhone, but we are currently dealing with some complications due to the limitations imposed by the iOS. Other directions for further experiments may concern the downloading task rather than the uploading one considered here. Nevertheless, our study opens a wide range of investigations and applications thanks to the innovative and intriguing approach that takes advantage of multi-interfaces. For instance, we believe that the coupling of 5G (and its device-to-device communication capability) together with 5GHz WiFi could indeed be rather effective in drone data collection activities for reducing the expensive hovering time.

## Figures and Tables

**Table 1:** Characteristics of the smartphones that have been used in our experiments.

	Android					Bluetooth			WiFi		Cellular
**Smartphone**	**6.0.1**	**7.1.1**	**7.1.2**	**8.1 Go**	**8.1**	**4**	**4.1**	**4.2**	**2.4 GHz**	**5 GHz**	**4G**
Nexus 5	X				X	X			X	X	X
Motorola G4		X					X		X		X
Samsung S4			X			X			X	X	X
Nokia N1				X				X	X		X

## References

[1] Ullah Z., Al-Turjman F., Mostarda L., Gagliardi R. (2020). Applications of Artificial Intelligence and Machine learning in smart cities. Comput. Commun..

[2] Al-Turjman F., Lemayian J.P., Alturjman S., Mostarda L. (2019). Enhanced Deployment Strategy for the 5G Drone-BS Using Artificial Intelligence. IEEE Access.

[3] Friedman R., Kogan A., Krivolapov Y. (2013). On Power and Throughput Tradeoffs of WiFi and Bluetooth in Smartphones. IEEE Trans. Mob. Comput..

[4] Cano C., Leith D.J. Coexistence of WiFi and LTE in Unlicensed Bands: A Proportional Fair Allocation Scheme. Proceedings of the 2015 IEEE International Conference on Communication Workshop (ICCW).

[5] Wang X., Quek T.Q.S., Sheng M., Li J. (2017). Throughput and Fairness Analysis of Wi-Fi and LTE-U in Unlicensed Band. IEEE J. Sel. A. Commun..

[6] Ickin S., Zinner T., Wac K., Fiedler M. Catching the download train: Energy-efficient file downloading on smartphones. Proceedings of the 2014 26th International Teletraffic Congress (ITC).

[7] Paasch C., Bonaventure O. (2014). Multipath TCP. Queue.

[8] Habak K., Harras K.A., Youssef M. (2015). Bandwidth aggregation techniques in heterogeneous multi-homed devices: A survey. Comput. Netw..

[9] Kaiping X., Ke C., Dan N., Hong Z., Peilin H. (2016). Survey of MPTCP-Based Multipath Transmission Optimization. J. Comput. Res. Dev..

[10] Abbas N., Hajj H.M., Dawy Z., Jahed K., Sharafeddine S. (2017). An optimized approach to video traffic splitting in heterogeneous wireless networks with energy and QoE considerations. J. Netw. Comput. Appl..

[11] Sharafeddine S., Jahed K., Fawaz M. (2017). Optimized device centric aggregation mechanisms for mobile devices with multiple wireless interfaces. Comput. Netw..

[12] Doddapaneni K., Ever E., Gemikonakli O., Malavolta I., Mostarda L., Muccini H. Path Loss Effect on Energy Consumption in a WSN. Proceedings of the 14th International Conference on Computer Modelling and Simulation, 2012.

[13] D’Angelo G., Di Stefano G., Navarra A., Ibrahiem M.M., El Emary S.R. (2013). Multi-Interface Wireless Networks: Complexity and Algorithms. Wireless Sensor Networks: From Theory to Applications.

[14] Bahl P., Adya A., Padhye J., Walman A. (2004). Reconsidering wireless systems with multiple radios. SIGCOMM Comput. Commun. Rev..

[15] Cavalcanti D., Gossain H., Agrawal D. Connectivity in multi-radio, multi-channel heterogeneous ad hoc networks. Proceedings of the 2005 IEEE 16th International Symposium on Personal, Indoor and Mobile Radio Communications.

[16] Faragó A., Basagni S. The Effect of Multi-Radio Nodes on Network Connectivity—A Graph Theoretic Analysis. Proceedings of the 2008 IEEE 19th International Symposium on Personal, Indoor and Mobile Radio Communications.

[17] Draves R., Padhye J., Zill B. Routing in multi-radio, multi-hop wireless mesh networks. Proceedings of the 10th International Conference on Mobile Computing and Networking (MobiCom).

[18] Caporuscio M., Charlet D., Issarny V., Navarra A. Energetic Performance of Service-oriented Multi-radio Networks: Issues and Perspectives. Proceedings of the 6th International Workshop on Software and Performance (WOSP).

[19] Klasing R., Kosowski A., Navarra A. (2009). Cost Minimization in Wireless Networks with a Bounded and Unbounded Number of Interfaces. Networks.

[20] Aloisio A., Navarra A. (2020). Budgeted Constrained Coverage on Series-Parallel Multi-Interface Networks. Proceedings of the 16th International Conference on Advanced Information Networking and Applications (AINA).

[21] Aloisio A., Navarra A. (2020). Budgeted constrained coverage on bounded carving-width and series-parallel multi-interface networks. Internet Things.

[22] Aloisio A., Navarra A. (2020). Constrained Connectivity in Bounded X-Width Multi-Interface Networks. Algorithms.

[23] Aloisio A., Navarra A., Mostarda L. (2020). Energy consumption balancing in multi-interface networks. J. Ambient Intell. Humaniz. Comput..

[24] D’Angelo G., Di Stefano G., Navarra A. (2012). Minimize the Maximum Duty in Multi-interface Networks. Algorithmica.

[25] Athanassopoulos S., Caragiannis I., Kaklamanis C., Papaioannou E. (2013). Energy-Efficient Communication in Multi-interface Wireless Networks. Theory Comput. Syst..

[26] Kosowski A., Navarra A., Pinotti M. (2010). Exploiting Multi-Interface Networks: Connectivity and Cheapest Paths. Wirel. Netw..

[27] Kosowski A., Navarra A., Pajak D., Pinotti C. (2013). Maximum matching in multi-interface networks. Theor. Comput. Sci..

[28] D’Angelo G., Di Stefano G., Navarra A. (2014). Flow problems in Multi-Interface Networks. IEEE Trans. Comput..

[29] Audrito G., Bertossi A., Navarra A., Pinotti C. (2017). Maximizing the overall end-user satisfaction of data broadcast in wireless mesh networks. J. Discret. Algorithms.

